# Plasma Cystatin C and High-Density Lipoprotein Are Important Biomarkers of Alzheimer’s Disease and Vascular Dementia: A Cross-Sectional Study

**DOI:** 10.3389/fnagi.2017.00026

**Published:** 2017-02-07

**Authors:** Rui Wang, Zhaoyu Chen, Yongmei Fu, Xiaobo Wei, Jinchi Liao, Xu Liu, Bingjun He, Yunqi Xu, Jing Zou, Xiaoyan Yang, Ruihui Weng, Sheng Tan, Christopher McElroy, Kunlin Jin, Qing Wang

**Affiliations:** ^1^Departments of Neurology, The Third Affiliated Hospital of Sun Yat-Sen UniversityGuangzhou, China; ^2^Departments of Emergency, The Third Affiliated Hospital of Sun Yat-Sen UniversityGuangzhou, China; ^3^Departments of Radiology, The Third Affiliated Hospital of Sun Yat-Sen UniversityGuangzhou, China; ^4^Department of Neurology, Nanfang Hospital, Southern Medical UniversityGuangzhou, China; ^5^Departments of Neurology, Zhujiang Hospital, Southern Medical UniversityGuangzhou, China; ^6^Department of Pharmacology and Neuroscience, University of North Texas Health Science CenterFort Worth, TX, USA; ^7^Guangdong Province Key Laboratory of Brain Function and DiseaseGuangzhou, China

**Keywords:** Alzheimer’s disease, vascular dementia, cystatin C, high-density lipoprotein, dementia

## Abstract

**Objectives**: Cystatin C (Cys C) and high-density lipoprotein (HDL) play critical roles in neurodegenerative diseases, such as dementia, Alzheimer’s disease (AD) and vascular dementia (VaD). However, whether they can be used as reliable biomarkers to distinguish patients with dementia from healthy subjects and to determine disease severity remain largely unknown.

**Methods**: We conducted a cross-sectional study to determine plasma Cys C and HDL levels of 88 patients with dementia (43 AD patients, 45 VaD patients) and 45 healthy age-matched controls. The severity of dementia was determined based on the Schwab and England Activities of Daily Living (ADL) Scale, the Mini-mental State Examination (MMSE), the Global Deterioration Scale (GDS), the Lawton Instrumental ADL (IADL) Scale, and the Hachinski Ischemia Scale (Hachinski). Receiver operating characteristic (ROC) curves were calculated to determine the diagnostic accuracy of Cys C and HDL levels in distinguishing patients with dementia from healthy subjects.

**Results**: We found that plasma Cys C levels were higher, but HDL levels were lower in AD and VaD patients respectively, compared to healthy control subjects. Yet, Cys C levels were highest among patients with VaD. Interestingly, plasma Cys C levels were significantly correlated with IADL Scale scores. In addition, the ROC curves for Cys C (area under the curve, AUC 0.816 for AD, AUC 0.841 for VaD) and HDL (AUC 0.800 for AD, AUC 0.731 for VaD) exhibited potential diagnostic value in distinguishing AD/VaD patients from healthy subjects. While the ROC curve for the combination of Cys C and HDL (AUC 0.873 for AD, AUC 0.897 for VaD) showed higher diagnostic accuracy in distinguishing AD/VaD patients from healthy subjects than the separate curves for each parameter.

**Conclusions**: Our findings suggest that the inflammatory mediators Cys C and HDL may play important roles in the pathogenesis of dementia, and plasma Cys C and HDL levels may be useful screening tools for differentiating AD/VaD patients from healthy subjects.

**Highlights:**

- Plasma Cys C levels were higher in patients with AD/VaD than in healthy subjects.
- Plasma HDL levels were lower in patients with AD/VaD than in healthy subjects.
- Plasma Cys C levels were significantly correlated with dementia.
- The ROC curve for the combination of Cys C and HDL showed potential diagnostic value in distinguishing AD/VaD from healthy subjects.

## Introduction

Dementia represents a broad category of brain diseases that usually cause declines in memory and gradual decreases in cognition that affect a person’s quality of life (Paulsen et al., 2013). Common types of dementia include Alzheimer’s disease (AD), vascular dementia (VaD), Lewy body dementia (LBD), frontotemporal dementia (FTD), multiple system atrophy dementia (MSA-D) and Parkinson’s disease dementia (PDD). Among these, AD and VaD are the most common types (Posada-Duque et al., 2014; Xu et al., 2014). AD is characterized by the accumulation of amyloid-β (Aβ) peptides and Tau (Kandimalla et al., 2011, 2014; Bourdenx et al., 2015; Hoppe et al., 2015). Meanwhile, VaD is characterized by the occurrence of minor strokes and the development of cognitive deficits (Ray et al., 2013). However, it is hard to differentiate from each other only based on the clinical features (Ray et al., 2013).

The cystatins (Cyss) are a family of cysteine protease inhibitors. Cys B and C have been increasingly investigated in neurological diseases such as AD and VaD. Cys B, a member of the cysteine protease inhibitor family, plays an important role in regulating abnormal accumulation of Aβ peptide and maintaining lipofuscin-related autofluorescence and giant lipid-containing autolysosomes in AD (Yang et al., 2011a,b, 2014; Boyle et al., 2013). Cys C, a potent cysteine inhibitor that is encoded by the Cys C (CST3) gene and that is secreted by all human tissues, is found in all body fluids (Heywood et al., 2015). It has been used as a biomarker of renal function as well as a strong predictor of VaD and AD (Kaur and Levy, 2012; Zhong et al., 2013). Multiple lines of evidence indicate that Cys C is functionally associated with anti-inflammation (Dutta et al., 2012; Jonsdottir et al., 2013) and exerts protective effects against age-related diseases (Xu et al., 2005; Mi et al., 2007; Kaur et al., 2010; Liu et al., 2014).

In AD and VaD, lipoproteins play crucial roles in preserving cognitive function (Ray et al., 2013; Ahmed et al., 2014; Dias et al., 2014). High-density lipoprotein (HDL) is part of a heterogeneous group of lipoprotein particles that exist in the systemic circulation and brain and mainly facilitate the clearance and delivery of lipids and lipid-related molecules from and throughout the body, respectively (Hottman et al., 2014). Several lines of evidence suggest that plasma HDL and its main protein component, apoA-I, also possess potent vasoprotective properties such as facilitating improvements in vascular function, inhibiting inflammation, suppressing endothelial reparation, preventing lipid oxidation, and stimulating endothelial repair (Stukas et al., 2014).

Both Cys C and HDL not only modulate dementia but also associate with vascular function and mediate vasorelaxation, inflammation, and oxidative stress (Dutta et al., 2012; Jonsdottir et al., 2013; Stukas et al., 2014). Therefore, we are interested in investigating the levels of Cys C and HDL in different types of dementia. To our knowledge there is a paucity of studies exploring the combined effects of Cys C and HDL in patients with dementia. In this study, we investigate whether Cys C and HDL are associated with the severity and prevalence of different types of dementia. The primary aim of this study was to compare plasma Cys C/HDL levels between patients with AD/VaD and healthy subjects. The secondary aim of this study was to explore the diagnostic value of plasma Cys C/HDL in dementia. Lastly, we also aimed to determine the correlations between the above plasma markers and the severity of dementia.

## Materials and Methods

### Patients and Ethics Statement

This cross-sectional study was performed at the Department of Neurology of the Third Affiliated Hospital Sun Yat-sen University, Guangzhou, China. From November 2012 to October 2015, a total of 88 patients with dementia (AD and VaD) were recruited for this study. Additionally, 43 patients with AD (20 males and 23 females) were enrolled, and their diagnoses were confirmed using the National Institute of Neurological and Communicative Diseases and Stroke-AD and Related Disorders Association (NINCDS-ADRDA) criteria for AD (McKhann et al., 1984). This study also included 45 patients with VaD (24 males and 21 females) whose diagnoses were confirmed using the National Institute of Neurological Disorders and Stroke-Association Internationale pour la Recherche et l’Enseignement en Neurosciences (NINDS-AIREN) criteria for VaD (Román et al., 1993). The MMSE scores of all patients were less than 25. A total of 45 healthy age-matched subjects (16 males and 29 females) were recruited from the outpatient setting and served as the control group. The control group was selected from the Medical Examination Centre of the Third Affiliated Hospital of Sun Yat-sen University. In this study, no subject presented with hypertension, cardiopathy, diabetes or renal dysfunction. In addition, none of the patients presented abnormal levels of the prostate carcinoma-related mediators prostate-specific antigen (PSA), carcinoembryonic antigen (CEA), and alpha-fetoprotein (AFP).

This study was approved by the ethics committee of the Third Affiliated Hospital of Sun Yat-sen University and was conducted according to the principles outlined in the revised Declaration of Helsinki of 1975 and the National Institutes of Health Human Subjects Policies and Guidelines released in 1999. All participants provided written consent to participate in the investigation and allowed researchers to measure their plasma Cys C and HDL levels. All subjects participated in the following standardized assessments: the Schwab and England Activities of Daily Living (ADL) Scale, the Webster Scale, the modified Hoehn and Yahr Staging Scale (H&Y), the Mini-mental State Examination (MMSE), the Global Deterioration Scale (GDS), the Lawton Instrumental ADL (IADL) Scale, and the Hachinski Ischemia Scale (Hachinski). Patients with different types of disease participated in different standardized assessments, which were all conducted in a blinded manner.

### Study Design

**Figure 1:**
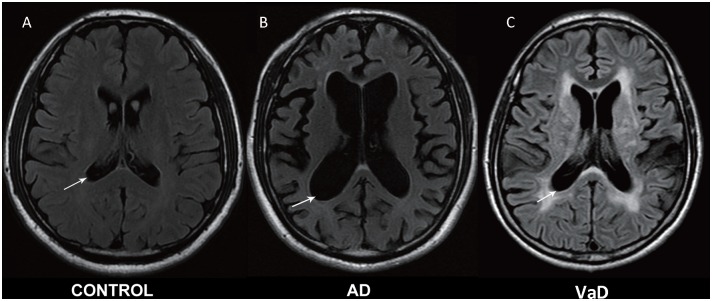
**Magnetic resonance imaging (MRI) images in normal control, Alzheimer’s disease (AD) and vascular dementia (VaD) patients. (A)** Healthy control. **(B)** Cerebral atrophy and enlargement of the cerebral lateral ventricles of AD patients. **(C)** Leukoencephalopathy and encephalomalacia foci of VaD patients. All MRI are shown in T2-weighted image.

Experienced neurologists were recruited to perform evaluations and complete neurological examinations of both the inpatients and the outpatients. All patients with AD met the NINCDS-ADRDA criteria, and all patients with VaD met the NINDS-AIREN criteria (Román et al., 1993). The following patients were excluded: (1) patients with physical disability due to neurological disorders other than AD or VaD such as congenital diseases or psychosis; (2) patients with somatic disabilities caused by trauma or other diseases that may affect ADL and IADL, including pain syndromes, advanced diabetes mellitus, malignancy, renal failure, hepatic failure, cardiopathy, severe anemia, or any other acute or chronic debilitating or life-threatening disease/state; (3) patients with MMSE scores higher than 25 (25 was included in the study); and (4) patients who refused to participate in the study. All subjects completed the following standardized assessments: the ADL Scale, IADL Scale and GDS, which stratified patients based on their clinical disabilities (Kaufman et al., 2013); Hachinski, which stratified patients based on whether they had VaD, AD or another type of dementia (AD < 4, VaD > 7); and the MMSE, which evaluated patient cognitive abilities. All scales were available and validated for the Chinese population. All subjects have been scanned by magnetic resonance imaging (MRI), and the typical MRI for normal control, AD and VaD patients are shown in Figure 1.

### Blood Sample Measurements

Five milliliters of venous blood was taken to measure Cys C and HDL levels. All measurements were repeated three times. Plasma was isolated by centrifugation at 3000 rpm for 10 min within 1 h of sample collection. The separated plasma was stored at −30°C before laboratory evaluation (Chen et al., 2015; Gao et al., 2015). Cys C levels were measured with a latex-enhanced reagent (N Latex Cys C, Dade Behring, Deerfield, IL, USA), using a Behring BN ProSpec Analyzer (Dade Behring). Plasma HDL levels were measured via direct enzymatic methods, using commercial kits (Cholestest N-HDL, Sekisui Medical, Tokyo, Japan) with less than 3% intra-assay and inter-assay coefficients of variance.

### Statistical Analysis

All continuous variables (age; MMSE, ADL, IADL, and GDS scores; and HDL and Cys C levels) were presented as the mean ± standard deviation if the data were normally distributed or as medians (min, max) if the data were not normally distributed. Categorical variables are presented as percentages. ADL and IADL scores were determined by summing individual items. To assess the significance of differences between groups, Student’s *t* test was applied when the data were normally distributed. Tukey’s *post hoc* analysis was conducted to compare differences in HDL and Cys C levels among normal subjects, according to gender. Pearson’s correlation (*r*_p_) and Spearman’s rank correlation (*r*_s_) coefficient were used to evaluate correlations between different clinical parameters for normal data and non-normal data, respectively. Receiver operating characteristic (ROC) analysis was conducted to assess the diagnostic value of specific clinical biomarkers (Cys C, HDL) with respect to identifying the abovementioned diseases. In addition, an ROC curve for the combination of Cys C and HDL was calculated via logistic regression analysis to determine the value of this combination in predicting disease progression. *p* values < 0.05 were deemed statistically significant, and SPSS 13.0 software (Chicago, IL, USA) was used for the statistical analyses.

## Results

### Patient Characteristics

This cross-sectional study included 43 AD patients (20 males [47%] and 23 [53%] females), 45 VaD patients (24 males [53%] and 21 [47%] females), and 45 healthy subjects (16 males [36%] and 29 females [64%]). The mean ages of the AD patients, VaD patients and normal controls were 67.35 ± 10.48, 69.11 ± 7.98 and 64 ± 6.47 years, respectively. Clinical evaluating parameters in AD and VaD were shown in Table 1. There was no significance difference in age between the patients and control subjects (AD vs. Control, *p* = 1.000; VaD vs. Control, *p* = 0.191; Student’s *t*-test). The demographic and clinical data for the subjects are shown in Tables 1A,B).

**Table 1A:** **Clinical parameters in Alzheimer’s disease (AD)**.

Variable	AD			AD M			AD F		
	Mean (SD)	Min	Max	Mean (SD)	Min	Max	Mean (SD)	Min	Max
MMSE	14.00	0	22	14.00	0	22	12.50	0	21
ADL (Barthel)	95.00	20	100	90.00	20	100	95.00	55	100
GDS	5.00	3	7	5.00	3	7	5.00	3	6
Hachinski	2.00	1	3	2.00	1	3	2.00	1	3
IADL	5.00	0	17	5.00	0	15	9.00	0	17

*Abbreviations: MMSE, Mini-mental State Examination; ADL, Schwab and England Activities of Daily Living Scale; GDS, Global Deterioration Scale; Hachinski, Hachinski Ischemia Scale; IADL, Lawton Activities of Daily Living Scale*.

**Table 1B:** **Clinical parameters in vascular dementia (VaD)**.

Variable	VaD			VaD M			VaD F		
	Mean (SD)	Min	Max	Mean (SD)	Min	Max	Mean (SD)	Min	Max
MMSE	16.10 (5.18)	3	24	15.29 (5.20)	3	23	17.19 (5.10)	6	24
ADL (Barthel)	82.50	20	100	87.50	20	100	75.00	20	100
GDS	4.00	2	6	4.00	3	5	4.00	2	6
Hachinski	11.00	7	14	9.50	7	14	11.00	7	13
IADL	13.13 (3.50)	6	21	13.13 (3.44)	6	21	13.38 (3.65)	4	19

*Abbreviations: MMSE, Mini-mental State Examination; ADL, Schwab and England Activities of Daily Living Scale; GDS, Global Deterioration Scale; Hachinski, Hachinski Ischemia Scale; IADL, Lawton Activities of Daily Living Scale*.

### Comparisons of Cys C/HDL Levels between Patients with Dementia and Healthy Subjects

**Figure 2:**
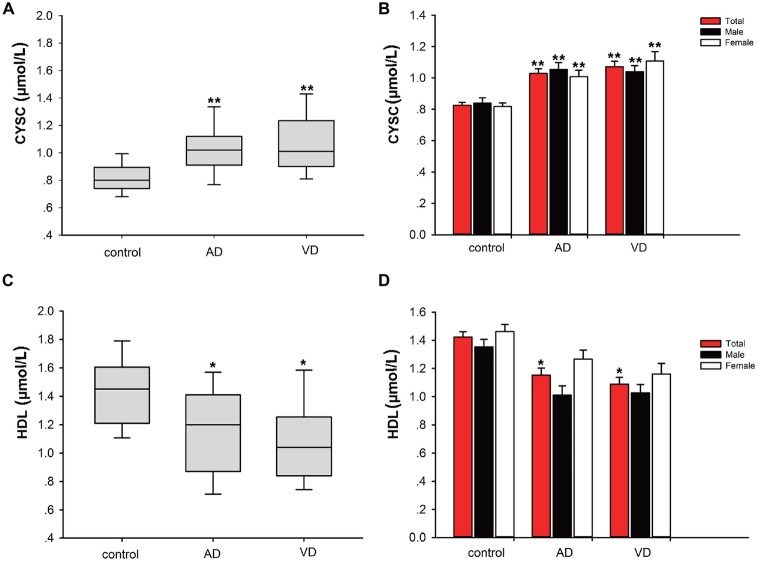
**Comparison of Cystatin C (Cys C) and high-density lipoprotein (HDL) levels between AD/VaD patients and control subjects. (A)** Comparison of Cys C levels between the dementia and control groups. **AD vs. control, *p* < 0.001; **VaD vs. control, *p* < 0.001. **(B)** Comparison of Cys C levels between the dementia and control groups according to gender. *AD (male) vs. control (male), *p* = 0.035; **AD (female) vs. control (female), *p* = 0.014; **VaD (male) vs. control (male), *p* = 0.043; **VaD (female) vs. control (female), *p* < 0.001. **(C)** Comparison of HDL levels between the dementia and control groups. *AD vs. control, *p* = 0.023; **VaD vs. control, *p* = 0.001. **(D)** Comparison of HDL levels between the dementia and control groups according to gender. No significant differences in HDL levels were found between the dementia (male) and control (male) and dementia (female) and control (female) groups.

In this study, significant differences in plasma Cys C levels were observed between patients with dementia (AD/VaD) and healthy subjects (Table 2). Plasma Cys C levels were higher in AD and VaD patients than in normal subjects (AD, 1.03 ± 1.94 vs. 0.83 ± 0.13, **p < 0.001; VaD, 1.07 ± 0.23 vs. 0.83 ± 0.13, **p < 0.001; Student’s *t*-test, (Tables 1A,B; Figure 2). Moreover, in patients with AD or VaD, HDL levels were significantly lower than in healthy subjects (AD: 1.15 ± 0.32 vs. 1.42 ± 0.25, *p = 0.023; VaD: 1.09 ± 0.32 vs. 1.42 ± 0.25, **p = 0.001, Student’s *t*-test, Table 1B; Figure 2).

**Table 2:** **Comparison of Cystatin C (Cys C), high-density lipoprotein (HDL), CREAT, blood urea nitrogen (BUN) and uric acid (UA) levels among AD patients, VaD patients and normal healthy subjects**.

Variable	Control Mean ± SD	AD Mean ± SD	VaD Mean ± SD	*p* value Control vs. AD	*p* value Control vs. VaD
CYSC	0.83 ± 0.13	1.03 ± 1.94	1.07 ± 0.23	<0.001**	<0.001**
HDL	1.42 ± 0.25	1.15 ± 0.32	1.09 ± 0.32	0.023*	0.001**
CREAT	69.00 ± 12.89	75.67 ± 13.76	77.41 ± 17.94	0.558	0.257
BUN	5.51 ± 1.33	5.57 ± 1.61	5.62 ± 1.92	1.000	1.000
UA	316.64 ± 59.28	345.43 ± 91.07	323.31 ± 93.84	0.772	1.000
Age	64 ± 6.47	67.35 ± 10.48	69.11 ± 7.98	1.000	0.191

*p < 0.05, **p < 0.01. Student’s t test.

### Correlations between Cys C and HDL Levels and MMSE, ADL, IADL and GDS Scores

We utilized correlation analysis to investigate the correlations between the abovementioned inflammatory mediators and various assessment tools. Depending on the data distribution, Pearson’s and Spearman’s correlations were used separately for different diseases to evaluate the correlations between disease severity and clinical variables (Tables 3A,B). In AD patients, there were significant correlations between Cys C levels and Hachinski scores (*r*_s_ = 0.349, *p = 0.022, Table 3A) and Cys C levels and age (*r*_s_ = 0.575, **p = 0.000, Table 3A). However, there were no significant correlations between Cys C/HDL levels and the scores of the other assessments. In VaD patients, a significant correlation was noted only between HDL and age (*r*_s_ = 0.403, **p = 0.006, Table 3B).

**Table 3A:** **Spearman’s rank correlation coefficient (*r*_s_) and *p* values pertaining to the relationships between clinical variables and MMSE, IADL, GDS, Hachinski and ADL scores in AD**.

Variable	AD (total)				AD (male)				AD (female)			
	CYSC		HDL-C		CYSC		HDL-C		CYSC		HDL-C	
	*r*	*p*	*r*	*p*	*r*	*p*	*r*	*p*	*r*	*p*	*r*	*p*
MMSE	0.069	0.658	0.110	0.479	−0.278	0.250	0.220	0.365	0.413*	0.045	0.041	0.851
IADL	−0.065	0.681	0.176	0.258	−0.464*	0.046	0.151	0.536	0.335	0.110	−0.033	0.877
GDS	0.036	0.820	−0.197	0.204	0.447	0.055	−0.216	0.375	−0.310	0.140	−0.193	0.366
Hachinski	0.349*	0.022	−0.002	0.989	0.060	0.806	−0.106	0.667	0.524**	0.009	0.034	0.875
ADL (Barthel)	−0.081	0.602	0.188	0.222	−0.368	0.121	0.098	0.689	0.157	0.463	0.142	0.507
Age	0.575**	0.000	0.042	0.788	0.619**	0.005	−0.015	0.952	0.540**	0.006	0.152	0.479

*p < 0.05, **p < 0.01. Abbreviations: r, Spearman’s rank correlation coefficient; MMSE, Mini-mental State Examination; ADL, Schwab and England Activities of Daily Living Scale; GDS, Global Deterioration Scale; Hachinski, Hachinski Ischemia Scale; IADL, Lawton Activities of Daily Living Scale.

**Table 3B:** **Pearson and Spearman’s rank correlation coefficient (*r*_s_) and *p* values pertaining to the relationship between clinical variables and MMSE, IADL, GDS, Hachinski and ADL scores in VaD**.

Variable	VaD (total)				VaD (male)				VaD (female)			
	CYSC		HDL-C		CYSC		HDL-C		CYSC		HDL-C	
	*r*	*p*	*r*	*p*	*r*	*p*	*r*	*p*	*r*	*p*	*r*	*p*
MMSE	0.036	0.816	0.150	0.326	−0.132	0.538	0.085	0.694	0.121	0.601	0.147	0.525
IADL	−0.028	0.854	0.045	0.781	0.014	0.948	0.195	0.361	−0.042	0.858	−0.246	0.282
GDS	−0.003	0.986	0.166	0.277	0.082	0.704	0.168	0.433	−0.010	0.967	0.199	0.388
Hachinski	0.121	0.430	−0.074	0.631	0.071	0.742	−0.133	0.537	0.216	0.348	−0.116	0.617
ADL (Barthel)	0.054	0.724	−0.016	0.919	0.149	0.487	0.236	0.267	−0.022	0.926	−0.178	0.440
Age	0.128	0.401	0.403**	0.006	−0.014	0.949	0.195	0.361	0.317	0.161	0.552**	0.009

*p < 0.05, **p < 0.01. Abbreviations: MMSE and IADL scale scores were analyzed via Pearson’s correlation; GDS, ADL scale scores and Hachinski scores were analyzed by Spearman’s rank correlation; MMSE, Mini-mental State Examination; ADL, Schwab and England Activities of Daily Living Scale; GDS, Global Deterioration Scale; Hachinski, Hachinski Ischemia Scale; IADL, Lawton Instrumental Activities of Daily Living Scale.

To eliminate the influence of confounders (e.g., gender), we divided the AD and VaD patients and the healthy subjects into two groups (females/males). In male AD patients, there were significant correlations between Cys C levels and age (*r*_s_ = 0.619, **p = 0.005, Table 3A) and Cys C levels and IADL scores (*r*_s_ = −0.464, *p = 0.046, Table 3A). Meanwhile, in female AD patients, there were significant correlations between Cys C levels and MMSE scores (*r*_s_ = −0.413, *p = 0.045, Table 3A) Cys C levels and Hachinski scores (*r*_s_ = 0.524, **p = 0.009, Table 3A), and Cys C levels and age (*r*_s_ = 0.540, **p = 0.006, Table 3A). However, in VaD patients, a significant correlation was observed only between HDL and age (*r*_s_ = 0.552, **p = 0.009, Table 3B).

### ROC Analysis of the Utility of Cys C and HDL Levels in the Diagnosis of Dementia

**Figure 3:**
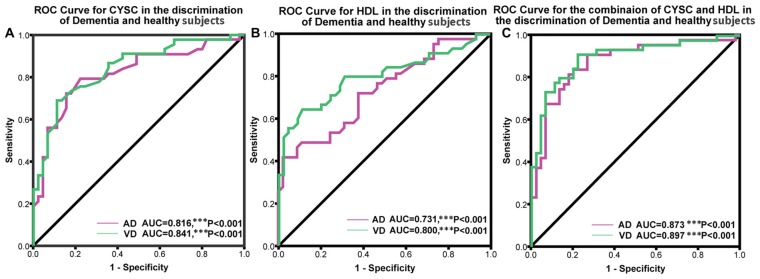
**Receiver operating characteristic (ROC) curves evaluating the utility of plasma levels of Cys C and HDL in distinguishing patients with dementia from healthy controls. (A)** The area under the curves (AUCs) of the ROC curves for Cys C in AD and VaD were 0.816 (95% CI: 0.724–0.908, ***p < 0.001) and 0.841 (95% CI: 0.627–0.835, ***p < 0.001). **(B)** The AUCs of the ROC curves for HDL in AD and VaD were 0.731 (95% CI: 0.759–0.923, ***p < 0.001) and 0.800 (95% CI: 0.705–0.894, ***p < 0.001). **(C)** The AUCs of the ROC curves for Cys C + HDL in AD and VaD were 0.873 (95% CI: 0.797–0.950, ***p < 0.001) and 0.897 (95% CI: 0.831–0.964, ***p < 0.001).

ROC analysis was conducted to determine the diagnostic value of Cys C and HDL, according to the area under the ROC curve (AUC). Using ROC analysis, we determined the capacity of the above markers to distinguish patients with dementia from normal subjects. We also analyzed Cys C and HDL levels in AD patients. The AUC for Cys C was 0.816 (95% CI: 0.724–0.908, ***p < 0.001); the cut-off was 0.91 μmol/L, with a sensitivity of 79% and a specificity of 78%. Similarly, the AUC for HDL was 0.731 μmol/L (95% CI: 0.759–0.923, ***p < 0.001); the cut-off was 1.04 μmol/L, with a sensitivity of 42% and a specificity of 98%. Furthermore, in VaD patients, the AUC for Cys C was 0.841 (95% CI: 0.627–0.835, ***p < 0.001); the cut-off was at 0.97 μmol/L, with a sensitivity of 81% and a specificity of 51%. The AUC for Cys C was 0.800 (95% CI: 0.705–0.894, ***p < 0.001); the cut-off was 1.14 μmol/L, with a sensitivity of 64% and a specificity of 89% (Figure 3).

In addition, we also conducted an ROC analysis for the combination of Cys C and HDL in different types of dementia. The AUC for AD was 0.873 (95% CI: 0.797–0.950, ***p < 0.001), with a sensitivity of 91% and a specificity of 73%, and the cut-off was 0.36, depending on the predicted risk algorithm. Meanwhile, the AUC for VaD was 0.897 (95% CI: 0.831–0.964, ***p < 0.001); the cut-off was 0.34, depending on the predicted risk algorithm, with a sensitivity of 91% and a specificity of 78%. These data suggest that the ROC curve for the combination of AD/VaD has a higher diagnostic value than the separate curves for uric acid (UA) or HDL alone in distinguishing AD and VaD patients from healthy subjects.

## Discussion

In the present study, we explored variations in plasma Cys C and HDL levels in AD and VaD and noted several interesting results. First, we observed a remarkable increase in plasma Cys C levels and a decrease in HDL levels in AD/VaD patients compared to healthy subjects. Second, we noted significant correlations between plasma Cys C levels and severity scores in AD and VaD patients. Third, ROC analysis strongly suggested that the combination of Cys C and HDL can significantly distinguish AD and VaD patients from healthy subjects and can therefore be used as a new screening and diagnostic instrument. The AUC increased after incorporating plasma Cys C and HDL levels. This means that the diagnostic accuracy of the combination of the two variables was superior to that of either variable alone in differentiating AD/VaD patients from healthy subjects. To our knowledge, this is the first study to explore variations in plasma Cys C and HDL levels in AD and VaD patients.

AD and VaD are neurodegenerative diseases with different etiologies and pathogeneses. Therefore, it is important to explore the underlying mechanisms of these diseases and the usefulness of specific biomarkers in evaluating their severity and progression. There is evidence that inflammatory responses may be associated with the pathogenesis of AD and VaD, and there are also some clinical similarities between AD and VaD as both diseases cause gradual impairments in cognition, follow a progressive course, and affect activities of daily living. Several lines of recent evidence show that both Cys C and HDL can act as anti-inflammatory mediators and exert neuroprotective effects against AD and VaD progression (Gauthier et al., 2011; Hottman et al., 2014; Stukas et al., 2014). However, whether Cys C and HDL can be used in the evaluation and diagnosis of AD and VaD remains unknown. Therefore, we examined whether these anti-inflammatory mediators can be employed as viable and reliable biomarkers in diagnosing AD and VaD and in assessing their severities. We also identified clinical variables that are associated with AD and VaD in different gender groups.

Although the present, Cys C’s function in the brain is unclear, but it has been implicated in both neuronal degeneration and nervous system repair. Enhanced CysC expression occurs in human patients and in animal models of neurodegenerative conditions (Liu et al., 2014). In our study, we noted significantly increased plasma Cys C levels in AD and VaD patients compared to healthy subjects. These results were consistent with previous reports. Similar results were observed in two previous cross-sectional studies that noted higher and lower plasma Cys C levels in AD and MCI mild cognitive impairment (MCI) patients, respectively, and an increasing but non-significant trend in these levels in AD patients compared to those in healthy subjects (Ghidoni et al., 2010; Sundelöf et al., 2010). This finding implies that plasma Cys C may play an important role in AD and VaD. However, different studies have reported lower CSF levels of Cys C in AD patients and no significant changes in plasma Cys C levels in AD patients compared to controls (Hansson et al., 2009; Zhong et al., 2013). Sundelöf et al. (2008) also indicated that low levels of plasma Cys C precede clinical AD in elderly men who are free of dementia at baseline and may be an indicator of future AD risk. We speculate that during the early stages of diseases such as MCI, Cys C fails to protect neurons from the toxicity of oligomeric Aβ, which contributes to AD (Kaur and Levy, 2012), while during the later stages of dementia, Cys C levels increase in response to disease progression to protect neurons from further damage. Differences in study design and a lack of correlation between CSF Cys C and plasma Cys C levels or GFR may explain the differences in the results of studies regarding Cys C and AD risk. Prospective studies regarding Cys C levels in CSF in relation to future AD risk are lacking. Thus, longitudinal studies of Cys C levels in CSF in relation to AD risk are needed (Sundelöf et al., 2010).

Low levels of HDL were associated with an increased risk for neuronal degeneration (Ward et al., 2010; Liu et al., 2014; Cai et al., 2016; Zhang et al., 2016). We noted decreased plasma HDL levels in AD and VaD patients compared to healthy subjects, implying that HDL contributes to the pathophysiological mechanisms underlying these disease processes. HDL-mediated anti-oxidative stress processes play an important role in the pathogenesis of AD and VaD. The precise mechanisms underlying the involvement of HDL in AD and VaD have not yet been elucidated, but we theorize that decreases in plasma HDL may exert vasoprotective effects, preserve cognitive function, and weaken anti-inflammatory responses in AD and VaD patients (Patterson and Holahan, 2012; Hottman et al., 2014; Stukas et al., 2014). Cys C and HDL are strongly correlated with vascular function (Kaur and Levy, 2012; Dias et al., 2014; Hottman et al., 2014; Villeneuve et al., 2014). Here, we observed that the plasma levels of Cys C and HDL were higher and lower, respectively, in VaD than in AD, respectively. This strongly suggests that vascular remodeling plays a more important role in VaD than in AD (Kaur and Levy, 2012; Dias et al., 2014; Villeneuve et al., 2014).

Numerous lines of evidence indicate that Cys C and HDL are associated with cognitive diseases (Kaur and Levy, 2012). In this study, we observed correlations between Cys C levels and Hachinski scores and age via Pearson’s correlation analysis. After dividing the subjects according to gender, we observed a positive correlation between IADL/MMSE sores and plasma Cys C levels in female AD patients. These results strongly imply that plasma Cys C levels can be used to assess cognitive dysfunction and quality of life in patients with dementia.

Because multiple lines of evidence support the neuroprotective roles of Cys C and HDL in neurodegenerative disorders and cognitive decline (Ghidoni et al., 2011; Hottman et al., 2014), we aimed to determine whether the combination of Cys C and HDL levels can be applied to distinguish AD and VaD patients from healthy subjects and to evaluate disease progression. To determine the diagnostic value of plasma Cys C and HDL in AD and VaD, we conducted an ROC analysis and noted an AUC of 0.816 for Cys C and an AUC of 0.731 for HDL in AD and an AUC of 0.841 for Cys C and an AUC of 0.80 for HDL in VaD. The AUCs for Cys C and HDL were all higher than 0.70 in AD and VaD, which means that both Cys C and HDL have high diagnostic value and an acceptable sensitivity and specificity for distinguishing AD/VaD patients from healthy subjects. Notably, Cys C and HDL were more reliable in distinguishing patients from healthy subjects in VaD than in AD (Figures 3A,B). Moreover, the combination of Cys C and HDL exhibited a better ability to distinguish between patients and controls than Cys C or HDL alone, with an AUC of 0.873 for AD and an AUC of 0.897 for VaD. The reliability and potential utility of the combination of Cys C and HDL as a diagnostic plasma biomarker in screening for AD and VaD is r eflected in our ROC analysis. Our findings have important clinical relevance. Using plasma Cys C and HDL levels as a screening tool, clinicians may be able to detect AD and VaD and screen for early disease in AD/VaD patients.

There were several limitations to our study that should not be ignored: (1) a small number of participants (43 AD patients, 45 VaD patients and 45 normal subjects) were recruited; (2) genetic factors, such as the CST3 and APOA1–2 genotypes, and anti-lipemic administration were not considered in this study; and (3) to validate and complete the questionnaire, we chose only AD and VaD patients with sufficient cognitive ability, which significantly narrowed the study population. The above limitations may have resulted in bias with respect to Cys C and HDL levels in AD and VaD patients. Therefore, it is necessary to conduct larger population studies in the future.

In summary, the findings of the current study support the idea that inflammation and vascular burden may contribute to the pathogenesis of AD and VaD. Of the biomarkers studied (Creatinine (Cr), blood urea nitrogen (BUN), UA, Cys C and HDL), Cys C and HDL were the most suitable and reliable anti-inflammatory mediators in distinguishing AD and VaD patients from healthy subjects and in evaluating disease progression. We speculate that higher Cys C levels and lower HDL levels may be associated with an elevated risk of dementia and may predispose patients to progressive disease. The anti-inflammatory effects of Cys C and HDL are important as they provide testable parameters that are related to the pathophysiology of AD and VaD. ROC curves, in combination with plasma Cys C and HDL levels, may be valuable for early diagnosis of AD and VaD and may be used to increase diagnostic accuracy with respect to differentiating AD and VaD patients from healthy subjects. Our findings support the use of plasma Cys C and HDL levels to diagnose AD and VaD, but neuropathological correlations may be required to confirm the diagnoses. To our knowledge, this is the first study to consider the combination of plasma Cys C and HDL levels in diagnosing and assessing the severity of AD and VaD. Additional studies are needed to determine whether plasma Cys C and HDL abnormalities are reliable parameters in distinguishing patients with dementia from healthy subjects, particularly during the early stages of disease.

## Author Contributions

RWang, ZC, YF, XW, JL, KJ and QW conceived and designed the clinical study; RWang, ZC, XW, YF, JL, JZ, XL, YX and QW performed the clinical study; RWang, JL, XY, ST, RWeng, BH and QW analyzed the data; BH and XL contributed reagents/materials/analysis tools; RWang, ZC, CM, KJ and QW wrote the article.

## Conflict of Interest Statement

The authors declare that the research was conducted in the absence of any commercial or financial relationships that could be construed as a potential conflict of interest.

## Abbreviations

AD: Alzheimer’s disease
ADL: Schwab and England Activities of Daily Living Scale
AUC: area under the curve
BUN: blood urea nitrogen
CNS: central nervous system
Cr: creatinine
Cys C: cystatin C
GDS: Global Deterioration Scale
Hachinski: Hachinski Ischemia Scale
HDL: high-density lipoprotein
H&Y: modified Hoehn and Yahr Staging Scale
IADL: Lawton Instrumental Activities of Daily Living Scale
MMSE: Mini-mental State Examination
NINCDS-ADRDA: National Institute of Neurological and Communicative Diseases and Stroke-Alzheimer’s Disease and Related Disorders Association
NINDS-AIREN: National Institute of Neurological Disorders and Stroke-Association Internationale pour la Recherche et l’Enseignement en Neurosciences
ROC: receiver operating characteristic
*r*_s_: Spearman’s rank correlation coefficient
*r*_p_: Pearson’s correlation coefficient
SD: standard deviation
UA: uric acid
VaD: vascular dementia.
